# Sexual behavior and experience of sexual coercion among secondary school students in three states in North Eastern Nigeria

**DOI:** 10.1186/1471-2458-6-310

**Published:** 2006-12-23

**Authors:** Ademola J Ajuwon, Adeniyi Olaleye, Banji Faromoju, Oladapo Ladipo

**Affiliations:** 1 African Regional Health Education Center, Department of Health Promotion and Education, College of Medicine, University of Ibadan, Nigeria; 2 Association for Reproductive and Family Health, Ibadan, Nigeria

## Abstract

**Background:**

Interest in the reproductive health of adolescents continues to grow throughout the world. Few studies had explored the reproductive health knowledge, sexual behavior and experience of sexual coercion among secondary school students in North Eastern states of Nigeria. The objectives of this descriptive survey were to collect data to plan appropriate interventions that meet the reproductive health knowledge, service and skills needs of students in Bauchi, Borno and Gombe states.

**Methods:**

Face-to-face interviews were conducted for 624 consenting students who were randomly selected from eighteen secondary schools using an 83-item structured questionnaire. Data were collected on demographic profile, reproductive health knowledge, sexual behavior and experience of sexual coercion.

**Results:**

The mean age of the respondents was 16.5 years. There were slightly more males (52%) than females (48%). Students' knowledge about reproductive health was generally low even though girls had better knowledge than boys. Thirteen percent of the entire students had had sexual experience; significantly more males (19%) than females (6%) had done so (p < 0.001).

Among boys the age at sexual debut ranged from 10–26 with a mean of 15.7 and median of 16. By contrast, the age at first sex among girls ranged from 10 to 18 years with a mean and median of 16.1 and 17 years respectively. Only 24% of those who were sexually active used a condom during their last sexual encounter. Overall 11% of the students reported that they had been tricked into having sex, 9% had experienced unwanted touch of breast and backside, and 5% reported rape.

**Conclusion:**

Students low reproductive health knowledge and involvement in risky sexual activities predispose them to undesirable reproductive health outcomes.

## Background

Interest in the reproductive health of adolescents continues to grow throughout the world. One of the factors responsible for this interest is the sheer number of young persons worldwide. For example, nearly half of the global population is less than 25 years old [1]. Addressing the reproductive health information and service needs of this population poses significant challenges for policy makers and service providers especially those working in resource poor settings. The fact that adolescents and other young persons are disproportionately affected by the reproductive health morbidity such as abortion, sexually transmitted infections (STI) including HIV/AIDS draws attention to the need of appropriate interventions. Adolescents account for a significant proportion of unsafe abortions globally. According to the World Health Organization (WHO) at least one-third of all women seeking hospital-care for abortion complications are under the age of 20 years [2]. Adolescents are also one of the groups hard hit by HIV/AIDS. Over half of all new HIV infections in Africa in 2005 were among young persons aged 14–25 years with the worst hit being young women [3]. These data underscore the need to target adolescents with appropriate interventions that address not only the contextual factors such as gender roles and poverty that place them at risk but also individual factors including lack of access to knowledge, inadequate communication and life skills that adolescents need to negotiate safe sex.

In Nigeria, as elsewhere in Sub-Saharan Africa, studies confirm that a large proportion of adolescents have unmet reproductive health needs. Evidence of unmet need is reflected in the fact that some adolescents and other young persons lack adequate knowledge and understanding of the reproductive process, that many harbor misconceptions such as the belief that mosquitoes can transmit HIV infection, and false claims that use of contraceptives can cause infertility [4,5]. The National HIV/AIDS and Reproductive Health Survey (NARHS) [6] showed that only 7% and 44% of 15–19 year olds knew of STI symptoms in men and women. Research also confirms that many young persons participate in risky sexual activities including early debut of sexual activities [7], multiple sexual partners, low and inconsistent use of condoms [5,8,9]. The data from the NARHS further reveal that among the sexually active 15 to 19 year olds only 34.4% used condoms at their most recent sexual encounter [7]. The explanations offered for increasing proportion of young persons involved in pre-marital sexual activities include earlier menarche, effects of mass media that glamorize sex, and increasing weakness of traditional control of the family system in Nigeria [10].

Although these researches have contributed to our understanding of the reproductive health behavior of young persons in Nigeria, the utility of the data is undermined by the fact that these studies were derived mainly from the southern parts of the country. Few studies have been conducted among young Nigerians living in the northern areas of the country. One recent study from Plateau, a predominantly Christian state in north central Nigeria, shows that many students participate in risky sexual activities including sexual networking and low use of condoms [8]. We are not aware of any published data on the reproductive health knowledge, sexual behavior and experience of sexual coercion among the adolescents from the northeastern region of Nigeria. This article is based on a study conducted among secondary school students in three states in this area. The objectives of the study were to collect data to plan appropriate interventions that meet the reproductive health knowledge, service, and skills needs of students living in this part of the country.

## Methods

### The setting

This study was a cross-sectional survey conducted in 2004 in Bauchi, Borno and Gombe, three of the six states of North Eastern Nigeria. As in most parts of northern Nigeria, Islam is the predominant religion in these states with some operating the Sharia legal system. In this area, early marriage is the norm and polygyny is widespread. Islamic law, which governs the ways of life of Muslims in the region, permits the marriage of girls who have not started menstruation. According to the National Demographic Health Survey of 2003, the median age at first marriage in North Eastern Nigeria is 14.7 years [11]. The major ethnic groups in the areas are Hausa, Fulani and Kanuri who live in major towns in the area including Bauchi, Maiduguri and Gombe, which also serve as the administrative capitals of the Bauchi, Borno and Gombe states respectively. Subsistence farming and fishing are the predominant occupations of citizens living in these states.

### Procedure

Established in 1989, the Association for Reproductive and Family Health (ARFH) is a national not-for profit non-governmental organization (NGO) located in Ibadan, Oyo state, with a track record of implementing good quality programs addressing the reproductive health needs of young persons, health workers, and women throughout Nigeria and elsewhere in Africa. ARFH conducted the study in collaboration with three youth-serving Non-Governmental Organizations (NGO), National Association of Nigerian Nurses and Midwives (NANNM) in Bauchi, Community Health and Youth Friendly Association (CHAYFA) in Borno and Guidance and Development Association (GCDA) in Gombe. Official permission to conduct the study was obtained from the policy makers in the States' Ministries of Health and Education and from the administrators in each of the schools that participated in the study.

### Sampling

Six public secondary schools from each state (total 18) were chosen for the survey. The schools were selected purposively with approval from the state Ministries of Education. A simple random technique was adopted in selecting students from each school. The first step in the process of selection was an enumeration of all arms (streams) in the junior and senior classes. Secondly, one arm was randomly selected by balloting. In each selected arm, the number of students present during the day of visit was divided into two male and female. Twenty students (20) were randomly selected by balloting from each of the junior and senior classes totaling forty (40) students in each school. In states where mixed schools were not available, single gender schools were selected such that appropriate number of males and females were recruited.

### Measures

An-83 item anonymous questionnaire containing open-ended and closed-ended questions was developed and used for data collection. The questionnaire built on previously used instrument for similar surveys among adolescents in Nigeria and Ghana [12]. The questionnaire was divided into four major sections for ease of administration. The demographic section focused on personal characteristics of respondents and their living situation, knowledge of reproductive health was explored by requesting students to answer a number of questions including definition of ovulation, safe period and whether or not a girl could become pregnant during the first sexual encounter and awareness of contraceptives methods. Questions about sexual behavior included whether or not respondents have ever had sex, age at first sex, number of partners, frequency of sex, use of condoms during the most recent encounter. Respondents were also asked if they had ever become pregnant or made someone pregnant. With respect to sexual coercion, respondents were asked if they had ever experienced any of the six sexually coercive behaviors derived from previous studies in this environment [13]. As recommended by Ellsberg and colleagues [14], we used behaviorally specific questions on coercion: Has any of the following ever happened to you? "someone touched your body against your will", "someone tricked you in order to have sex with you", someone tried to force you to have sex", "someone forced you to have sex", "someone beat you for refusing to have sex with him/her" "someone gave you drugs in order to have sex with you".

### Data collection

In each state, five trained research assistants conducted face-to-face interviews with students. The training lasted about eight hours and the contents consisted of data collection procedures, interview techniques and interpersonal skills with special focus on adolescent reproductive health issues. During the training, the investigators reviewed each question in the questionnaire to ensure that the interviewers were familiar with them. At the end of the training, skills of the research assistants were verified to be adequate through role-play and return demonstration. Face-to-face interview was preferred to self administered method because field experiences with surveys of young people in Nigeria show that the former usually yield better rate of response and produce good quality data than the latter [6]. The interviews were conducted in English or Hausa (the language widely spoken in the study area) to ensure good comprehension. In order to avoid gender bias in responses, the interviewers were of the same sex with the respondents. Privacy was ensured by conducting the interviews in unoccupied separate classrooms or under the trees in quiet environments free from distractions. Verbal informed consent was received from each respondent by explaining the purpose of the study, that participation in it was voluntary, and that information provided will be kept confidential.

### Data analysis

The completed questionnaires were collated and entered into the computer. The data was analyzed with the Statistical Package for Social Sciences (SPSS) computer software and the results were presented in simple percentages. Analysis was stratified by sex to show how responses to the variables of knowledge, sexual behavior and coercion differ for males and females. Descriptive statistics was used to profile the study population. Logistic regression was then used to explore the demographic variables associated with reproductive knowledge, sexual experience and coercion.

## Results

### Demographic profile

The profile of the 624 respondents interviewed is shown in Table 1. The respondents' ages ranged from 10 to 28 years, with a mean of 16.5. The majority (53%) belonged to 15–19 years age group. However, a greater proportion of females (66%) than males (41%) belonged to 15–19 years age group. Slightly more males (57%) than females (55%) were in junior classes. Overall 60% of the students were from co-educational schools, but more females (72%) than males (48%) belonged to this type of school. With respect to religious affiliation, the majority of both male (54%) and female (47%) were Moslems. Forty-four percent of the entire sample reported that they had ever worked to earn money. However, a higher proportion of male (55%) than female (32%) had ever done so. Almost equal number of male (83%) and female (80%) were working for money at the time of the survey.

### Knowledge about reproductive health issues

The proportion of students who had correct knowledge about selected reproductive health issues by gender is shown in Table 2. Overall, female students had higher levels of knowledge of reproductive health variables than male students. For example, significantly more girls (59%) than boys (43%) knew that a girl could become pregnant at first sexual experience (p < 0.01); 66% of girls knew of at least one contraceptive compared to 58% of boys (p < 0.01). More girls (40%) than boys (24%) had heard of contraceptive pills. However, more boys (53%) than girls (51%) had heard of male condom (p > 0.01) and a higher proportion of boys than girls knew that a mosquito could not transmit HIV infection (43% vs. 34%) (p > 0.01).

### Sexual behavior

**Figure 1:**
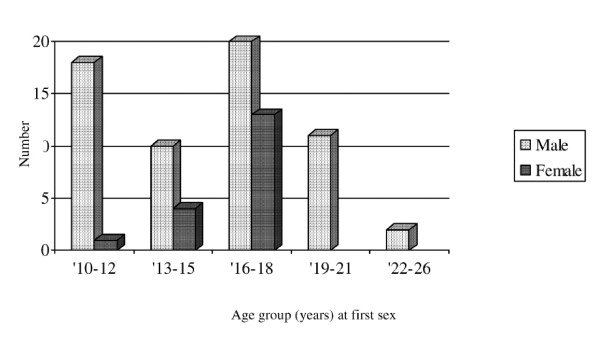
Age at first sex of secondary school students by gender.

The sexual behavior pattern of the students is shown in Table 3. Half (50%) of the entire students reported that they had ever had a romantic relationship with someone else. More females (59%) than males (43%) reported being involved in such relationship. Overall, only 13% of all the students reported that they had ever had sex. Significantly more males (19%) than females (6%) had ever had sex (p < 0.001). The mean age of first sex of the entire sexually experienced students was 15.8 years. The ages of first sex among boys and girls is shown in Figure 1. Among boys the age at sexual debut ranged from 10–26 with a mean of 15.7 and median of 16. More boys than girls had had sexual intercourse by age 16–18 years. By contrast, the age at first sex among girls ranged from 10 to 18 years with a mean and median of 16.1 and 17 years respectively. Concerning risk of exposure to HIV and other sexually transmitted infections, overall, only 24% of sexually experienced students reported that they used a condom during their last sexual encounter; however more females than males reported that they had done so. Fifteen percent of those who reported sexual experience had ever become pregnant or made someone to be pregnant; more females (33%) than males (10%) had ever done so.

Table 4 shows the relationship between demographic characteristics and sexual activities. Nineteen percent of students in Bauchi were sexually active compared to 13% and 10% of those in Borno and Gombe respectively (p < 0.001). Students from boy's only schools were significantly more likely to have had sexual intercourse (18%) than those from the co-educational (14%) and girl's only schools (2%) (p < 0.001). Similarly significantly more students who had ever worked for money (17%) were sexually active than those who had not (10%) (p < 0.001).

### Experience of sexual coercion

The proportion of students who reported any experience of sexual coercion is shown in Table 5. Overall, 36% of the entire students reported that they had experienced at least one of the six forms of sexually coercive behaviors explored in this study. Unwanted touch of the body (breast and backside) topped the list (31%) of the most frequently reported sexually coercive behavior which was followed by the attempt by someone to force the student to have sex (11%) and being tricked into having sex (9%). The proportion of students who report rape was 5% and the proportion was similar between the sexes.

### Logistic regression results

Logistic regression analysis was used to estimate/measure the effects of selected socio-demographic variables on three outcomes namely: sexual activity, sexual coercion and reproductive health knowledge. The selected variables included in the models were: state, type of school, location of school, sex, age, class, religion, living arrangement, having a boy/girl friend, ever worked for money and ever smoke cigarette. A respondent was considered to have reproductive health (RH) knowledge if he/she answered correctly at least three out of the eight knowledge questions asked; sexually active if he/she had ever had sexual intercourse while; sexual coercion was measured by experience of at least one of the listed forms of sexual coercion.

### Predictors of reproductive health knowledge

Major determinants of reproductive health knowledge were: state, sex, class and having boy/girl friends. The results showed that respondents in Gombe state were more likely to have knowledge of reproductive health issues than those in Bauchi and Borno states. Respondents in senior classes, females and those who had boy/girl friends were found to have higher odds of reproductive health knowledge. The results from logistic regression showed that with 95% confidence, respondents in junior classes were about 4 times more likely to have RH knowledge than those in senior classes, females were about 3 times more likely to have RH knowledge than males, while those who had boy/girl friends were 2 times more likely to have RH knowledge than those who did not have boy/girl friends (Table 6).

### Predictors of sexual activity

The result of the logistic regression showed that the major predictors of sexual activity were: type of school, location of the school, sex, age, living arrangement, religion and having a boy/girl friend. With 95% confidence, the odds of sexual activity were higher for respondents in co-educational schools, whose schools were located in urban areas, who had boy/girl friends, males and females. Specifically, respondents in co-educational schools were more likely to have had sexual intercourse than those in single sex schools. Respondents whose schools were located in urban areas were 3 times more likely to have had sexual intercourse than those in the rural schools while those in semi-urban schools were almost 2 times more likely to have had sex than students in rural areas. Males were 5 times more likely to be sexually active than females. Muslims respondents were less likely than Christians and other religion practitioners to have had sexual intercourse. The respondents who were living with persons other than their parents were more likely to have had sexual intercourse than those living with their parent(s). Similarly, the respondents who had boy/girl friends were 6 times more likely to have had sexual intercourse than those who did not have boy/girl friends (Table 6).

### Predictors of sexual coercion

Eight factors were found to significantly determine exposure to sexual coercion among the respondents. These factors were: state, type of school sex, age, class, religion, living arrangement and having boy/girl friends. The analysis showed that respondents in Bauchi and Borno states were 3 times and 6 times (respectively) more likely to have experienced sexual coercion than respondents in Gombe state. Ironically, males were more likely to have experienced sexual coercion than females. For a one year increase in age of respondents, the odds in favour of sexual coercion increased by a multiplicative factor of 0.97. The respondents in junior classes were almost 2 times less likely to experience sexual coercion than those in senior classes. Christian and Muslim respondents were less likely to experience sexual coercion than respondents who belonged to other/traditional religions. Respondents who were not living with their parent(s) were more likely to be exposed to sexual coercion than those who were living with their parent(s). Respondents who had boy/girl friends were 4 times more likely to experience sexual coercion than those who did not have boy/girl friends.

## Discussion

Adolescents are generally thought to be healthier than other age groups having survived many of the diseases of early childhood and being decades away from the diseases associated with aging [15]. However, some adolescents are affected by diseases while others die prematurely. A major threat to the health of the adolescents stems primarily from their sexual behavior which is partly influenced by lack of knowledge of reproductive health issues. Many of the students surveyed had limited knowledge of human reproduction. For example, only half of the students knew that pregnancy could occur during sexual debut. This finding is generally consistent with results from earlier studies in Nigeria in which many students have inadequate knowledge of reproductive health [6,16]. One survey showed that secondary school students had an average of 2.3 points on a 34 point reproductive health knowledge score [16]. Many Nigerian adolescents have poor understanding of the timing of conception; others harbor misconceptions such as the belief that a slipped condom can cause stomach discomfort and infertility [5-7].

Our findings show that females generally had higher knowledge of reproduction than males. One possible explanation may be the fact that because girls are disproportionately affected by the burden of reproductive health morbidity (STI, unwanted pregnancy, abortion), they are more likely than boys to seek for information about reproductive health. Also, parents are more likely to discuss reproductive health issues with girls than boys [6] because of the belief in Nigeria that boys will learn somehow through experimentation.

Overall, 13% of the students were sexually experienced. This figure is lower than the 34% reported among high school students from Plateau state, 62% of secondary school students in Ilorin, Kwara state [17] both of which are located in North central Nigeria [8], 48% of adolescent school girls in Lagos south western area [18], and 55% of students in Anambra and Enugu states in Southeastern Nigeria [5]. More importantly, significantly more males than females reported sexual activities, a finding consistent with previous surveys among young persons in this environment [5]. However, these data must be interpreted within the context of local culture in which the study took place and the methodological challenges associated with collecting data on sexual behavior from young persons. The apparently relatively low level of sexual activities among the surveyed students may be due to the fact that both Islamic and cultural sexual norms forbid premarital sexual activity which is a justification for the encouragement of early marriage in these areas.

At the same time these data may be an underestimation of actual levels of sexual activities because of the face-to-face interview method used in this study. As Plummer and colleagues [19,20] had pointed out, collection of valid data on sexual behavior from young persons is fraught with several methodological challenges including problems of recall, ambiguous terminology, and the sensitive nature of sexual information. Self-reported data may be invalid if respondents tell researchers what they believe is socially desirable, whether or not it is true, a bias that is likely to occur among adolescents who may be concerned about peer stigma or adult punishment [20]. Thus, it is possible that males may have over-reported their sexual activities and females under-report sexual activities in an attempt to conform to religious and cultural norms affecting sex in Northern Nigeria. Previous studies have shown that data from the self-completion questionnaire are likely to be more valid than those obtained from face-to-face interview because a greater sense of confidentiality for the respondent may reduce social desirability bias [21]. Yet, the self-completed method of data collection typically yields lower response rates and more missing data than the face-to-face method. Some researchers have recommended use of assisted self-completed questionnaire which addresses both of these limitations. The suitability of such a method in the Nigerian context will be explored in future studies.

There is concern that only about 4% of the sexually active students used a condom during their most recent sexual activities. Male condoms have been widely promoted in Nigeria as an efficient means of preventing the sexual transmission of HIV; condoms are relatively cheap, readily available, and do not require medical supervision. Yet, they remain under-utilized by many adolescents in Nigeria [22]. Some of the barriers to use of condoms by young Nigerians include misconceptions that a slipped condom may injure a girl [4]; lack of skills to negotiate its use with partners [22] and believe that use of condom suggests lack of trust or infidelity. On the other hand, female condoms are available in the country, but their exorbitant cost is a disincentive for its widespread use.

Experience of sexual coercion was a common phenomenon among the population surveyed. Overall, 5.1% of the students had been raped, a figure comparable to 4% found among female apprentice tailors [22] and to 6% in female hawkers operating in truck and bus stations in urban areas [23]. However the figure is lower than 8.6% reported among South African pupils [24]. Although these figures are relatively low, the data must be interpreted with caution since rape and other sexually coercive behaviours are typically under-reported due to stigma attached to this type of behaviour in Nigeria [23]. Contrary to results from previous surveys in Nigeria [22], South Africa [24] and Kenya [25] more males than females in this study reported experience of sexual coercion. One possible explanation may be the religious context in which the survey was conducted. In many parts of northern Nigeria women are kept in *purdah, i.e*. exclusion and when it is absolutely necessary for them to leave the home such as visiting a health facility or attend school they put on a veil and are not expected to have any direct contact with males. This may serve as protective factor. However, more studies need to be conducted in the future to have better understating of this phenomenon.

### Program implications

The results of this study provide baseline data for implementing appropriate interventions to address the reproductive health needs of students from these areas. One of the major findings of this study is that males tend to lag behind their female counterparts in each of the three main reproductive health issues namely knowledge, sexual behavior and coercion, explored in this study. The implication is that gender-based interventions would be required to address both the needs of males and female students. Three interventions are recommended to address the problems. First, gender-sensitive training of male and female students as peer educators is suggested to close gaps in students' knowledge about human reproduction. Peer education is appropriate because of its flexibility; for example trained educators can reach their colleagues whenever and wherever the topic comes up: in school, at home, street corners, and in shops [26]. The second strategy is the development of Youth Friendly Centers that would cater for the service needs relating to treatment of STI, provision of condoms and other non-prescriptive contraceptives. Such centers should preferably be located within the school environment or in other sites accessible to students and managed by trained staff that would provide comprehensive and appropriate services for young persons. Finally, teachers and parents have important roles to play in meeting the reproductive health needs of students. For practical purposes teachers would serve as role models and provide supportive supervision for trained peer educators. In addition, they would need to integrate reproductive health issues into relevant subjects in an attempt to reinforce information provided by peer educators. This is particularly necessary since reproductive health education in not included in the curricular in secondary schools in these states. The collaborative intervention provided by both students and teachers are likely to produce greater multiplier effects that would effectively address misconceptions and positively influence risky sexual activities [5].

This study has two limitations that must be pointed out. The first is the relatively small sample size selected from each state. Therefore the data must be interpreted with caution as they may not be generalized to the entire students' population in each state. Despite these limitations, the study is important because it represented the first attempt to systematically collect data on young person's reproductive health knowledge and sexual behavior in these areas. As such it has provided several valuable data for implementing intervention programs to meet the reproductive health information, services and skills needs of young people from these areas.

## Conclusion

In conclusion, young persons surveyed generally had low levels of knowledge and understanding of reproductive health issues. Many of those who are sexually active did not take precautions that would have protected them from the undesirable consequences of risky sexual activities. Interventions including peer education, training of teacher's ad development of youth friendly centers are recommended to meet the reproductive health needs of adolescents in the north eastern region of the country.

## Competing interests

The author(s) declare that they have no competing interests.

## Authors' contributions

AJA conceived the project, developed draft instruments, supervised data collection and wrote drafts of findings. AO collected data, performed analysis, and wrote draft manuscripts. BF developed draft instrument, wrote draft manuscripts. OAL conceived of project, wrote draft manuscript. All authors read and approved of final manuscript.

## Pre-publication history

The pre-publication history for this paper can be accessed here:



## Figures and Tables

**Table 1:** Demographic Characteristics of the Respondents by Gender

Variable	Male (N = 327)	Female (N = 297)	Total (N = 624)
**Age**			
10–14	73 (22)	89 (30)	162 (26)
15–19	133 (41)	195 (66)	328 (53)
20–24	74 (23)	3 (1)	77 (12)
25 and above	9 (2)	0	9 (1)
NA	38 (12)	10 (3)	48 (8)
**Class**			
Junior	186 (57)	163 (55)	349 (56)
Senior	141 (43)	134 (45)	275 (44)
**Type of school**			
Boys only	169 (52)	0	169 (27)
Girls only	0	84 (28)	84 (13)
Co-educational	158 (48)	213 (72)	371 (60)
**Location of school**			
Urban	100 (31)	116 (39)	216 (34)
Rural	88 (26)	116 (39)	204 (33)
Semi-urban	139 (43)	65 (22)	204 (33)
**Religion**			
Christianity	131 (40)	124 (42)	255 (41)
Islam	178 (54)	141 (47)	319 (51)
Others	18 (6)	32 (11)	50 (8)
**Living situation**			
Lived with both parents	275 (84)	228 (77)	503 (81)
Lived with a single parent	39 (12)	56 (18)	95 (15)
NA	13 (4)	13 (5)	26 (4)
**Had ever worked to earn money**			
Yes	180 (55)	95 (32)	275 (44)
No	147 (45)	202 (68)	349 (56)
**Currently working for money**			
Yes	147 (83)	76 (80)	223 (81)
No	33 (18)	19 (20)	52 (19)

The % are in brackets

**Table 2:** Proportion of students with correct knowledge of reproductive health by gender

Reproductive health Knowledge variable	Male (N = 327)	Female (N = 297)	Total (N = 624)	Statistics
A girl can be pregnant at first sexual experience	140 (43%)	175 (59%)	315 (50%)	p < 0.001*
Safe period in a woman menstrual cycle	22 (7%)	68 (23%)	90 (14%)	p < 0.001*
Had heard of any contraceptive	191 (58%)	196 (66%)	387 (62%)	P < 0.001*
Heard of a male condom	174 (53%)	150 (51%)	324 (52%)	p > 0.001
Heard of a female condom	22 (7%)	32 (11%)	54 (9%)	p > 0.001*
Heard of pills	80 (24%)	119 (40%)	199 (32%)	p < 0.001*
Knew that consistent & correct use of condom can prevent HIV infection	218 (67%)	206 (69%)	424 (68%)	p > 0.001
Knew that mosquito bite cannot spread HIV	140 (43%)	100 (34)	240 (39%)	p > 0.001

* The differences are significant

**Table 3:** Pattern of sexual behavior among secondary school students by gender

Variable	Male (N = 327)	Female (N = 297)	Total (N = 624) %
Ever had girl/boyfriend	140 (43%)	175 (59%)	315 (51%)
Had ever had sex	63 (19%)	18 (6%)	81 (13%)
Frequency of sex in last three months	(N = 63)	(N = 18)	
None	11 (17%)	0	11 (14%)
Once	15 (24%)	6 (33%)	21 (12%)
Two or more times	37 (59)	12 (67%)	59 (74)
Number of partners in last three months	(N = 52)	(N = 18)	
One	19 (37%)	11 (61%)	30 (43%)
Two or more	23 (44%)	3 (17%)	26 (37%)
None	10 (9%)	4 (22%)	14 (23%)
Used a condom a condom at last sex*	10 (16%)	5 (28%)	15 (24%)
Had ever become pregnant or made someone pregnant*	6 (10%)	6 (33%)	12 (15%)

* These refer to those who are sexually active

**Table 4:** Characteristics associated with sexual activities among students

Variables		Ever Had Sex		**P value**
			
		Yes (%)	No (%)	
**State**	Bauchi	*29 (19)*	*123 (81)*	p < 0.001*
	Borno	21 (13)	146 (87)	
	Gombe	31 (10)	273 (90)	
**Type of School**	Boys only	30 (18)	139 (82)	p < 0.001**
	Girls only	2 (2)	83 (98)	
	Co-educational	49 (13)	320 (87)	
**Location of School**	Urban	31 (14)	185 (86)	**p > 0.001**
	Semi-urban	28 (11)	227 (89)	
	Rural	*22 (14)*	*129 (86)*	
**Present Class**	Junior class	40 (16)	216 (84)	p > 0.001**
	Senior class	41 (18)	188 (82)	
**Gender**	Male	63 (19)	264 (81)	p < 0.001**
	Female	18 (6)	279 (94)	
**Religion**	Christianity	47 (18)	208 (82)	**p > 0.001**
	Islam	29 (11)	242 (89)	
	Others	5 (11)	45 (89)	
**Age**	10–14	7 (4)	155 (96)	p < 0.001**
	15–19	39 (12)	321 (89)	
	20–24	23 (30)	54 (66)	
	25 and above	2 (22)	7(78)	
	NA	10 (21)	38 (79)	
**Ever worked for money**				
**Yes**		46 (17)	229 (83)	p < .001*
**No**		35 (10)	314 (90)	

The % are in brackets

**Table 5:** Proportion of students who had experienced sexual coercion by gender

	Male	Female	TOTAL
Report any form of coercion	120 (37%)	104 (35%)	224 (36%)
Unwanted touch body	100 (31%)	93 (31%)	193 (31%)
Someone tried to force you to have sex	39 (12%)	30 (10%)	69 (11%)
Someone tricked you	37 (11%)	21 (7%)	58 (9%)
Someone forced you to have sex	19 (6%)	13 (4%)	32 (5%)
Someone beat you to have sex	10 (3%)	5 (2%)	15 (2%)
Someone gave you drugs to have sex	7 (2%)	2 (1%)	9 (1%)

**Table 6:** Odds from Logistic Regression Models of Reproductive Health Knowledge, Sexual Activity and Sexual Coercion by Selected Socio-demographic Variables

Explanatory Variables	Ever had sex C I	Experience of sexual coercion CI	Knowledge of SRH CI
State:			
Bauchi	1.60 (0.59–3.12)	2.26**(1.63–5.63)	0.55 (0.18–1.68)
Borno	1.37 (0.51–2.89)	5.66**(3.33–9.90) **	0.14** (0.02–.53) **
Gombe (RC)			
Type of School:			
Boys only	0.67 (0.32–1.16)	0.76 (.44–1.23)	0.89 (0.33–2.26)
Girls only	0.24* (0.03–0.92)	1.82 (1.00–4.20)	0.91 (0.20–4.10)
Co-educational (RC)			
Location of school:			
Urban	2.80** (1.42–7.45)	1.06 (0.65–2.20)	0.96 (0.32–3.98)
Semi-urban	1.54 (0.59–3.17) *	1.29 (0.73–2.52)	1.56 (0.72–4.28)
Rural (RC)			
Sex:			
Male	5.01** (2.44–10.24)	1.97**(1.10–2.82)	0.34** (0.16–.74)
Female (RC)			
Age	1.02 (0.99–1.03)	0.97** (0.93–.99)	1.01 (.98–1.04)
Present class:			
Junior secondary	1.10 (0.55–1.63)	0.60**(1.17–2.54)	0.24** (2.05–8.15)
Senior secondary (RC)			
Religion:			
Islam	0.58 (0.14–1.45)	0.35** (0.16–.67)	0.64 (0.15–1.64)
Christianity	1.38 (0.37–3.39) **	0.77 (0.36–1.45) **	0.92 (0.24–2.28)
Others (RC)			
Living arrangement:			
Living with both parents	0.54 (0.30–1.53)	0.56* (0.33–1.03)	0.70 (0.29–1.35)
Living with one parent	0.73 (0.24–2.70)	0.47* (0.22–1.27)	0.95 (0.16–2.94)
Living with others persons (RC)			
Ever had boy/girl friend (Yes)	6.30** (3.17–11.22)	4.19**(2.83–6.40)	2.46** (1.21–4.64)
Ever worked for money (Yes)	0.34 (0.71–2.12)	1.03 (0.66–1.46)	1.68 (0.89–3.10)
Ever Smoke cigarette (Yes)	0.00	2.10	0.01

** = P < .05; * = P < .10; RC = Reference Category

(95% confidence interval (CI) are in brackets)
